# Supplementary material for: Microbiological quality and genotoxicity of domestic water sources: A combined approach using Micro Biological Survey method and mutagenesis assay (micronucleus test) in root tips of Vicia faba in the West region of Cameroon
Source: PLoS One. 2021 Feb 4;16(2):e0245379. doi: 10.1371/journal.pone.0245379 (PMC7861400; doi:10.1371/journal.pone.0245379)
Supplement: Attachment — Submitted filename: Response to Reviewers.doc [file pone.0245379.s002.doc]

**A point-by-point response letter**

**Responses to general comments**

We are not the first authors of the laboratory protocols used in this study. We have added the references of these protocols in the main manuscript to allow reproducibility of the results.

A marked-up copy of the manuscript highlighting changes made to the original version was prepared and uploaded separately. An unmarked version of the revised paper without tracked changes was also submitted.

Our financing statement remains unchanged.

**Academic Editor**

**Journal Requirements:**

1. Please ensure that your manuscript meets PLOS ONE's style requirements, including those for file naming.

**Author’s responses:** We revised the manuscript according to PLOS ONE style requirements, including file naming.

2. In your Methods section, please provide additional details regarding the source of the water samples. Please provide the geographic coordinates of the collection locations to ensure reproducibility of the analyses.

**Author’s responses:** A table with sampling location names, water source categories, geographic coordinates and date of sample collection was added in the methods section.

3. We note that [Figure(s) 1] in your submission contain [map/satellite] images which may be copyrighted. All PLOS content is published under the Creative Commons Attribution License (CC BY 4.0), which means that the manuscript, images, and Supporting Information files will be freely available online, and any third party is permitted to access, download, copy, distribute, and use these materials in any way, even commercially, with proper attribution. For these reasons, we cannot publish previously copyrighted maps or satellite images created using proprietary data, such as Google software (Google Maps, Street View, and Earth).

**Author’s responses:** We are the authors of the figure 1; we used free software to generate the figure and details on software were added in the first revised manuscript, but we cannot provide other additional information regarding the licence, so we chose to delete figure 1 from the manuscript. GPS coordinates were provided in the manuscript for the results replication.

4. Please ensure that you refer to Figure 3 in your text as, if accepted, production will need this reference to link the reader to the figure.

**Author’s responses:** The correction has been done. There was confusion between tables and figures.

5. Please include a copy of Table 2 and 3 which you refer to in your text on page 17 and 18 (there are two tables uploaded named as Figure 2 and Figure 3).

**Author’s responses:** The correction was done.

6. Your ethics statement must appear in the Methods section of your manuscript. If your ethics statement is written in any section besides the Methods, please move it to the Methods section and delete it from any other section. Please also ensure that your ethics statement is included in your manuscript, as the ethics section of your online submission will not be published alongside your manuscript.

**Author’s responses:** The ethics statement was added in the Methods section and included in the manuscript.

7. We note that you have indicated that data from this study are available upon request. PLOS only allows data to be available upon request if there are legal or ethical restrictions on sharing data publicly. For information on unacceptable data access restrictions, please see http://journals.plos.org/plosone/s/data-availability#loc-unacceptable-data-access-restrictions.

**Author’s responses:** We change our declaration; the data sets used and/or analysed during the current study are available without restrictions.

8. In your revised cover letter, please address the following prompts:
a) If there are ethical or legal restrictions on sharing a de-identified data set, please explain them in detail (e.g., data contain potentially identifying or sensitive patient information) and who has imposed them (e.g., an ethics committee). Please also provide contact information for a data access committee, ethics committee, or other institutional body to which data requests may be sent.

**Author’s responses:** There are no ethical or legal restrictions on sharing data set. Data can be viewed without restrictions.

b) If there are no restrictions, please upload the minimal anonymized data set necessary to replicate your study findings as either Supporting Information files or to a stable, public repository and provide us with the relevant URLs, DOIs, or accession numbers.

**Author’s responses:** We changed our Data Availability statement and we will upload the study data set as Supporting Information files.

**Additional Editor Comments**

The article titled “Microbiological quality and genotoxicity of domestic water sources: A combined approach using Micro biological Survey method and mutagenesis assay (micronucleus test) in root tips of Vicia Faba in the West Region of Cameroon” is interesting, however, it has very high similarity with a previous article on "Microbiological quality of water sources in the West region of Cameroon: quantitative detection of total coliforms using Micro Biological Survey method". This article cannot be accepted in the present form and need Major revision in terms of presentation, methodology, and discussion. The manuscript lacks many technical details that require clarification and/or improvement. Authors are suggested to address the reviews comments in order to make the manuscript suitable for publication and provide point-by-point justification or rebuttal against the comments:

**Author’s responses:** Here, we provide a point-by-point response to the comments made by reviewers.

In the first study "Microbiological quality of water sources in the West region of Cameroon: quantitative detection of total coliforms using Micro Biological Survey method" we applied the MBS method as simple and rapid test to check water potability. In this second study, we combined the MBS and micronucleus test using *Vicia faba* seeds to propose an approach for bacteriological and chemical routine quality check of drinking water which can be easily implemented in developing countries.

**Comments to the Author**

1. Is the manuscript technically sounds, and do the data support the conclusions?

The manuscript must describe a technically sound piece of scientific research with data that supports the conclusions. Experiments must have been conducted rigorously, with appropriate controls, replication, and sample sizes. The conclusions must be drawn appropriately based on the data presented.

Reviewer #1: No

Reviewer #2: Partly

**Author’s responses:** All experiments conducted in this study were done according the standard's protocols (citations added in the main manuscript). We have further commented on our results to support our conclusion.

2. Has the statistical analysis been performed appropriately and rigorously?

Reviewer #1: No

Reviewer #2: Yes

**Author’s responses:** Statistical analyses were done using Stata IC/15.0 software (College Station, Texas 77845 USA, [http://www.stata.com](http://www.stata.com/)), and the main results were presented and interpreted.

3. Have the authors made all data underlying the findings in their manuscript fully available?

The PLOS Data policy requires authors to make all data underlying the findings described in their manuscript fully available without restriction, with rare exception (please refer to the Data Availability Statement in the manuscript PDF file). The data should be provided as part of the manuscript or its supporting information or deposited to a public repository. For example, in addition to summary statistics, the data points behind means, medians and variance measures should be available. If there are restrictions on publicly sharing data—e.g. participant privacy or use of data from a third party—those must be specified.

Reviewer #1: Yes

Reviewer #2: Yes

**Authors responses:** Nothing to add

4. Is the manuscript presented in an intelligible fashion and written in standard English?

PLOS ONE does not copyedit accepted manuscripts, so the language in submitted articles must be clear, correct, and unambiguous. Any typographical or grammatical errors should be corrected at revision, so please note any specific errors here.

Reviewer #1: No

Reviewer #2: No

**Author’s responses:** The manuscript was submitted to an English-speaking person for language revision and improves its quality.

5. Review Comments to the Author

Please use the space provided to explain your answers to the questions above. You may also include additional comments for the author, including concerns about dual publication, research ethics, or publication ethics. (Please upload your review as an attachment if it exceeds 20,000 characters).

**Authors responses:**

**Reviewer reports**

**Reviewer #1**

The work has been found to be very interesting; however, there are some major suggestions with the respect to the design of the experiment and also discussions in the write-up:

1) Details about the sampling to be given in more depth

**Author’s responses:** More details in the sampling procedure of water sources were added in the manuscript. Water was collected (in the morning before 12 o’clock), we didn’t apply a statistical sampling method to identify water source to be collected. The sources of water were identified step by step according to their availability, accessibility, and their use as drinking water by the local populations.

2) As the microbial load of the water is to be tested, the time of the day or even the temperature at the time of collection is crucial, please provide the data

**Author’s responses:** The date and time of water sampling were provided in the manuscript. Temperature and pH of water were measured before leaved the site. The temperatures and pH didn’t significatively vary between water sources. The averages values of both measurements were given in the results section.

3) Why E. coli, please explain the need for enumeration of the indicator, depth in literature lacking especially in the introduction and discussion

**Author’s responses:** We didn’t enumerate E. coli but Total Coliforms bacteria which include Escherichia coli (major species in the faecal coliform group). The MBS test doesn’t permit to clearly identify the bacteria present in sample water. We provided the justification of using TC as water pollution indicator in the manuscript.

4) Why an enzymatic assay like MBS, why not membrane filtration? Explicitly mention the advantages and the underlying principle and the rationale behind the choice of the method

**Author’s responses:** We mentioned in the main manuscript the advantages of MBS and cited previous studies which applied this test for water analysis in Africa and in Cameroon.

5) Micronucleus test is a standard method, please cite where it has been adapted from

**Author’s responses:** We provided the citation in the text.

6) Flowchart on the protocol for micronucleus test is recommended

**Author’s responses:** We described all the steps of the MN analysis in the Methods section and we cited the source of the protocol. We think that these are sufficient for the results reproducibility.

7) 22 samples may be too few to come up with a valid conclusion

**Author’s responses:** As we mentioned above, the sources of water to be sampled were identified step by step according to their availability, accessibility. It is also important to note that we are in context of water scarcity.

**Reviewer #2**

**Comments:** The manuscript highlights the poor quality of domestic water sources in the Central African sub-region. The study deals with the microbial and genetic toxicity induced factors responsible for deteriorating water quality of different natural/domestic sources of drinking water. While this is worthy of investigation, the manuscript lacks in clarity of presentation, especially the methodology and discussion section. The manuscript provides a good read on detection of total coliforms (TC) which was done using the Micro Biological Survey-HACCP & water Easy test along with micronucleus test to assess the genetic toxicity caused due to the poor water quality. However, the approach of the study lacks many technical details that need clarification and/or improvement. Authors should improve the usage of the English language while revising the manuscript. Authors are suggested to address following comments in order to make the manuscript suitable for publication and provide point-by-point justification or rebuttal against each comment.

**Author’s responses:** We did our best to address the comments and improve the quality of the manuscript.

1. The content of abstract requires modification. Authors are suggested to make the abstract more clear and informative. Provide concise and factual abstract briefly and sequentially stating the aims (objectives), major results, and principal conclusions of the work. Derive the conclusion at last. Provide the data (values) for significant findings of the present study

**Author’s responses:** The abstract was revised as recommended.

2. Abstract, line 29, and Study design and period, line 123, At each site,…genotoxic analysis. The word ‘litter’ should be replaced by ‘litre’ which is a unit of volume

**Author’s responses:** Done

3. Introduction, lines 78-80, According to World Health Organization…between life stages. Please rephrase the sentence

**Author’s responses:** Done

4. Total coliforms detection, Lines 130-131, what does it mean by rapid color change? Specify the changes in color corresponding to a definite range for the determination of increasing or decreasing coliform count.

**Author’s responses:** A rapid color change (from red to yellow) indicates a very high concentration of coliforms in the water sample. We rephrased the sentence. The change in color according to the time of analyses and the corresponding concentrations of coliforms are given in the main manuscript.

5. Total coliforms detection, Lines 130-131, which reagent is the author talking about? Please mention

**Author’s responses:** We are talking about test, not a reagent. The MBS kit comes in a pack containing all the material necessary for the analysis without need additional reagent. More details on MBS-HACCP & water Easy test are given in the main manuscript.

6. Total coliforms detection, Lines 141-145, please mention the specific color change and concentration range (very high, high, and low) for coliform count after 24 hours. Also clarify the reason for the reducing coliform counts with the passage of time.

**Author’s responses:** There is one change in color from red to yellow (contamination). The results were interpreted according to the standard protocol. We specified the level of Coliforms concentration according to the time required for the color change. 12h = very high contamination; 19h = high contamination and 24h = low contamination. The color change is proportional to the concentration of coliforms in the water samples, the greater the number of microorganisms, the more rapid the change of color, thus, a positive result (contamination).

7. Total coliforms detection, lines 141-145, A color change…103 CFU/ml. Please rephrase the sentence.

**Author’s responses:** Done

8. Of the 4 water sources sampled (as mentioned in study design and discussion), results of drilling water is not mentioned in Table 1. Further, results lack the complete details on TC concentration range along all the sources. Also, provide the coordinates for the sampling locations.

**Author’s responses:** Results of drilling water were mentioned; we used a synonym, now we cleared mentioned drilling water in all tables. We provide all result details available when using MBS method. The most important result is the indication of whether TC bacteria are present or not and the level of concentration. The coordinates of sampling locations were provided in the manuscript.

9. The reason for opting/choosing Vicia faba as a study plant is not mentioned in the methodology. Authors need to explain with proper rationale or any background study.

**Author’s responses:** The reasons for opting/choosing Vicia faba were given in the Methods section.

10. MN (micronucleus) test abbreviation must be given in the sub headings rather than providing details within the text body for better understanding of readers. Also brief introduction about MN test is not provided as given for Micro Biological Survey-HACCP & water Easy test for coliforms. Please include.

**Author’s responses:** Done

11. The major criticism of the manuscript lies in the lack of technical details provided for the methodology adopted. Authors are suggested to make this section clearer and explanatory. How well water is supposed to be more contaminated with higher coliform count during rainy reason and not in the surface water source? Explanation provided is not satisfactory and subject to contradiction. More meaningful and concrete justification is required

**Author’s responses:** Additional details on the methods used were given, and we specified (and cited) that we used the standard protocols. The analyses used in this study are simple to execute and don’t require sophisticated instruments or additional reagent (MBS method). We provide all the steps of our methodology necessary for the reproducibility of the results.

We are talking about concentration (quantity) of bacteria in well water. A previous study conducted in the same area (Bafoussam), during the same season found similar results. We think that bacteria growth is most high in groundwater than surface water (running water), and the combined effect of temperatures (from 22 to 26 °C) and pH (5.5 to 8.3) could favour bacteria growth. The proximity between wells and latrines could also favour contamination (by runoff water or slick phreatic communication). We added other justifications in the manuscript.

12. Discussion, TC concentration detected highest among wells, but later genotoxicity effects has been reported in spring water, river water as well as well water. What could be the possible reason for such differences? Authors need to address the same.

**Author’s responses:** There is no relation between genotoxicity and TC concentration, and results are coherent. If genotoxic effect is detected in water this indicates the presence of chemicals (disinfectant, heavy metal, etc.), and generally there are toxic for bacteria or limit bacteria growth (low concentration). Finally we noted in the study that some wells were chlorinated (that’s why 1 ml of Thiosulphate was added to water sample to neutralize the chlorine), so it’s consistent if genotoxic effects are also detected in well water.

13. What specific genotoxicity effects do author referring to?? Whatever genetic changes/ mutation detected in the experiments is nowhere mentioned in the manuscript. Please clarify.

**Author’s responses:** Micronucleus as mutagenesis endpoint (chromosome fragments). We clarified in the manuscript.

14. Discussion, line 215, “However, the TC concentration of untreated source water was found to be higher than treated water sources with no significant difference” – the sentence itself showing significant differences in TC concentration. Please rephrase suitably.

**Author’s responses:** No statistically significant difference at the significance level set. We rephrased.

15. Discussion, lines 217 – 219, (Other standardized……..environmental pollutions) – past studies and future need for more such studies needs to be differentiated well. Rephrase the sentence carefully.

**Author’s responses:** Done

16. Discussion, line 232, intensive agricultural activities instead of “intensives”. Please check the whole manuscript for other grammatical errors

**Author’s responses:** Done

17. The conclusion section must state the key findings of the study. Authors are suggested to draw major inferences/primary conclusions first from the results followed by the secondary conclusions reached through the critical analysis.

**Authors responses:** Done
