# Supplementary material for: Microbiological quality and genotoxicity of domestic water sources: A combined approach using Micro Biological Survey method and mutagenesis assay (micronucleus test) in root tips of Vicia faba in the West region of Cameroon
Source: PLoS One. 2021 Feb 4;16(2):e0245379. doi: 10.1371/journal.pone.0245379 (PMC7861400; doi:10.1371/journal.pone.0245379)
Supplement: Attachment — Submitted filename: Response to Reviewers.doc [file pone.0245379.s003.doc]

**Rebuttal letter**

**Additional Editor Comments (if provided):**

The article has been improved; however few miner corrections are required before publication as suggested by the reviewers.

**Author’s responses**: Corrections as suggested by the reviewers have been done (see below).

**Reviewers' comments**:

**Reviewer's Responses to Questions**

**Comments to the Author:**

1. If the authors have adequately addressed your comments raised in a previous round of review and you feel that this manuscript is now acceptable for publication, you may indicate that here to bypass the “Comments to the Author” section, enter your conflict of interest statement in the “Confidential to Editor” section, and submit your "Accept" recommendation.

Reviewer #1: All comments have been addressed

Reviewer #2: All comments have been addressed

**Author’s responses**: nothing to report

2. Is the manuscript technically sound, and do the data support the conclusions?

The manuscript must describe a technically sound piece of scientific research with data that supports the conclusions. Experiments must have been conducted rigorously, with appropriate controls, replication, and sample sizes. The conclusions must be drawn appropriately based on the data presented.

Reviewer #1: Partly

Reviewer #2: Yes

**Author’s responses**: We added more information in the revised manuscript as requested by Reviewer #1

3. Has the statistical analysis been performed appropriately and rigorously?

Reviewer #1: Yes

Reviewer #2: Yes

**Author’s responses**: nothing to report

4. Have the authors made all data underlying the findings in their manuscript fully available?

The [PLOS Data policy](http://www.plosone.org/static/policies.action#sharing) requires authors to make all data underlying the findings described in their manuscript fully available without restriction, with rare exception (please refer to the Data Availability Statement in the manuscript PDF file). The data should be provided as part of the manuscript or its supporting information, or deposited to a public repository. For example, in addition to summary statistics, the data points behind means, medians and variance measures should be available. If there are restrictions on publicly sharing data—e.g. participant privacy or use of data from a third party—those must be specified.

Reviewer #1: No

Reviewer #2: Yes

**Author’s responses**: We already change our data availability statement: Data used for this study are fully available without restriction and the data set was uploaded as supporting information during the revision process.

5. Is the manuscript presented in an intelligible fashion and written in standard English?

PLOS ONE does not copyedit accepted manuscripts, so the language in submitted articles must be clear, correct, and unambiguous. Any typographical or grammatical errors should be corrected at revision, so please note any specific errors here.

Reviewer #1: Yes

Reviewer #2: Yes

**Author’s responses**: nothing to report

6. **Review Comments to the Author**

Please use the space provided to explain your answers to the questions above. You may also include additional comments for the author, including concerns about dual publication, research ethics, or publication ethics. (Please upload your review as an attachment if it exceeds 20,000 characters)

**Author’s comments**:

It’s not a dual publication. As we already responded, in the study "Microbiological quality of water sources in the West region of Cameroon: quantitative detection of total coliforms using Micro Biological Survey method" we applied the MBS method as simple and rapid test to check water potability. In this second study, we combined the MBS and micronucleus test using Vicia faba seeds to propose an approach for bacteriological and chemical routine quality check of drinking water which can be easily implemented in developing countries.

Regarding the research ethics, or publication ethics, our manuscript does not report on or involve the use of any animal or human data or tissue, so an ethics approval and consent are not required with reference to Order No. 079/A/MSP/DS of the Minister of Public Health of October 22, 1987 establishing and organizing an Ethics Committee on Research Involving Human Beings (article 2).

**Reviewer #1:**

The work done is interesting however some suggestions on improving the clarity of the writeup is recommended:

1. Line 130: 1 mL of what concentration of thiosulphate? Is it sodium thiosulphate, if yes please specify, if not please mention the salt name explicitly

**Author’s responses**: It is 1 ml of Sodium Thiosulfate solution 10% (0.25g/50ml). We specified in the revised manuscript.

2. Line 133: Mention explicitly the temperature and pH were taken before or after placing the samples in the ice box

**Author’s responses**: Done: Temperature and pH of water were measured on site before placing the samples in the ice box. We mentioned explicitly it the revised manuscript.

3. Table 1: The temperature mentioned is for the water or ambient air temperature? Also, mentioning the time of collection is suggested

**Author’s responses**: The temperature mentioned is for the water samples, we specified it in the revised manuscript. We did not accurately record the time of sample collection, but as mentioned in the revised manuscript, sample collection was done in the morning before 12 o’clock.

4. Line 160-164: Add the reference

**Author’s responses**: Done!

5. For the section of total coliform detection: The test details insufficient, which enzymes play a crucial role in the color change? More details on the mechanism of color change needs to be explained to understand how the kit works?

**Author’s responses**: MBS method is a colorimetric reaction system based on the measurement of the catalytic activity of oxidoreductase enzymes of primary metabolism, which allows you to determine a correspondence between microorganism and enzyme activity in the sample. This precision was added in the revised manuscript.

6. Line 219: Why so much variation in the pH, was it because the samples were collected at different time frames of the day, was the time of collection not kept constant? Or was there appearance or disappearance of algal blooms?

**Author’s responses**: Some factors can explain the large variation of the water pH, such as time of samples collection. But think that we have minimized this influence since all the samples were collected in the same period (in the morning before 12 o'clock). Rather, we think that this variation of the pH may be due to the difference in depth of the water sources (wells, water drilling) or to the composition of the underground rock. We will take these factors into account in future studies.

Reviewer #2: The manuscript has been revised in accordance with the comments provided. Authors have also satisfactorily answered and clarified the doubts raised. The revised manuscript is now suitable for acceptance and publication in the Journal.

**Author’s responses**: nothing to report

7. PLOS authors have the option to publish the peer review history of their article ([what does this mean?](https://journals.plos.org/plosone/s/editorial-and-peer-review-process#loc-peer-review-history)). If published, this will include your full peer review and any attached files.


If you choose “no”, your identity will remain anonymous but your review may still be made public.

**Do you want your identity to be public for this peer review?** For information about this choice, including consent withdrawal, please see our [Privacy Policy](https://www.plos.org/privacy-policy).

Reviewer #1: No

Reviewer #2: No

**Author’s responses**: nothing to report
